# Graphene Composite via Bacterial Cellulose Assisted Liquid Phase Exfoliation for Sodium-Ion Batteries

**DOI:** 10.3390/polym15010203

**Published:** 2022-12-31

**Authors:** Kewei Shu, Siyu Tian, Yu Wang, Guiqiang Fei, Liyu Sun, Huizhu Niu, Yihao Duan, Guangyu Hu, Haihua Wang

**Affiliations:** 1 Xi’an Key Laboratory of Advanced Performance Materials and Polymers, Shaanxi Key Laboratory of Chemical Additives for Industry, Shaanxi University of Science and Technology, Xuefu Road, Weiyang District, Xi’an 710021, China; 2 Department of Materials Science and Engineering, Korea University, Seoul 02841, Republic of Korea; 3 College of Chemical Engineering, Shaanxi Institute of Technology, Xi’an 710300, China

**Keywords:** liquid phase exfoliation, bacterial cellulose, graphene composite, sodium-ion battery

## Abstract

One of the most critical challenges for commercialization of sodium-ion battery (SIB) is to develop carbon anodes with high capacity and good rate performance. Graphene would be an excellent SIB anode candidate due to its success in various kinds of batteries. Liquid-phase exfoliation (LPE) method is an inexpensive, facile and potentially scalable method to produce less-defected graphene sheets. In this work, we developed an improved, dispersant-assisted LPE method to produce graphene composite materials from raw graphite with high yield and better quality for SIB anode. Here, bacterial cellulose (BC) was used as a green dispersant/stabilizer for LPE, a “spacer” for anti-restacking, as well as a carbon precursor in the composite. As a result, the carbonized BC (CBC)/LPE graphene (LEGr) presented improved performance compared to composite with graphene prepared by Hummers method. It exhibited a specific capacity of 233 mAh g^−1^ at a current density of 20 mA g^−1^, and 157 mAh g^−1^ after 200 cycles at a high current density of 100 mA g^−1^ with capacity retention rate of 87.73%. This method not only provides new insight in graphene composites preparation, but also takes a new step in the exploration of anode materials for sodium-ion batteriesSIBs.

## 1. Introduction

Rechargeable lithium-ion batteries have already been remarkably employed for consumer electronics, electric vehicles and grid energy storage. However, the emerging problems, such as the scarcity of lithium resources and resulting soaring prices, need to be urgently addressed. At present, sodium ion batteries (SIBs) have gained increasing attraction due to their low cost, abundant sodium resources (2.3 wt% of Na vs. 0.002 wt% of Li), and similar battery electrochemistry [1,2,3,4]. However, a larger radius of Na^+^ than Li^+^ leads to lower ion diffusion coefficient and sluggish kinetics during intercalation/de-intercalation, causing poor electrochemical performance in SIBs [5]. In order to promote the commercialization of SIBs, a key challenge is to explore inexpensive and high-performance electrode materials, especially anode materials.

In particular, carbon materials are hopeful anode materials for SIBs [6,7,8], albeit there is a huge difference in selection criteria between SIB and LIB because of inherent differences in ion charge carriers. Graphitic carbon, which is widely used in LIB, exhibited poor electrochemical performance in SIB, which urges researchers to explore alternative carbon materials [9]. Graphene has excellent electrical conductivity, ultra-high light transmittance, superb mechanical strength, and has already demonstrated excellent performances in Li, Na, and K ion batteries [10,11,12,13]. David et al. prepared reduced graphene oxide (rGO) at various anneal temperatures for application as an anode material in SIBs. In this study, excellent cycling stability was achieved by rGO annealed at 500 °C, with a specific capacity of 140 mAh g^−1^ at 100 mA g^−1^ [14]. To date, the chemical oxidation-exfoliation is the mainstream approach for graphene production [15], but it has faced drawbacks, such as induced defects, toxic reagents, and complicated procedure. A non-oxidized, less defected graphene can be obtained by liquid-phase exfoliation (LPE) of graphite in proper solvent under a mild condition, such as ultrasonication or shear force [16]. Although the LPE method seems superior for easy production of composite ink and potential scalability [17], yet it still suffered from low yield, poor reproducibility, and, in some cases, long exfoliation time. In this regard, numerous methods have been developed to promote exfoliation efficiency by screening solvents and introducing a stabilizer/dispersant.

Nanocellulose possesses several fascinating features including low density, high surface area, good mechanical properties, and sustainability. The presence of both a hydrophilic -OH group and hydrophobic -CH moieties endow nanocellulose with amphiphilicity, which allows it to serve as a green dispersant for carbon nanomaterials [17,18]. Moreover, various types of nanocellulose, such as cellulose nanocrystal, nanofibrillated cellulose, and cellulose nanofibrils, were explored to exfoliate or disperse two-dimensional materials in aqueous medium [17,18,19]. For instance, Xu et al. developed a cellulose nanofibril (CNFs)/ TEMPO aqueous assisted exfoliation of graphite, yielding a stable dispersion with a graphene concentration of 1 mg mL^−1^ [18]. Yu et al. found that graphite can be exfoliated in MCC NaOH/urea solution to obtain a high concentration dispersion of 8.54 mg/mL by sonication-assisted LPE. The thickness of the graphene nanosheets obtained ranged from 0–6 nm [20]. Bacterial cellulose (BC) is a kind of nanocellulose with the characteristics of high aspect ratio, excellent mechanical properties, and good hydrophilicity [21,22]. Given the high abundance of hydroxyl groups, graphene sheets can be stabilized by green material via forming hydrogen bonds [23,24], thus preventing agglomeration between graphene sheets. Thus, BC has the potential to be an efficient exfoliation reagent to produce graphene with better quality and higher yield, as well as to help to overcome the limitation of the practical application of various graphene-based SIB anode materials [25,26].

In this work, the BC-assisted LPE method was used to obtain graphene nanosheets from bulk graphite. The obtained BC/liquid exfoliation graphene (LEGr) composite was then subject to carbonization without separation to form graphene composite materials for a sodium-ion battery. BC can act as the main exfoliation reagent, stabilizing the graphite flakes through the conjugation of hydroxyl groups and π electrons. The carbonized BC and few layer graphene assembly provides more capacitive sodium storage sites, which effectively enhances the electrochemical performance. The composite displayed excellent rate performance and good cycling stability, and it outperformed the electrochemical aspect from counterpart with graphene produced by Hummers method. The specific capacity of LEGr composite was 233 mAh g^−1^ at a current density of 20 mA g^−1^ and 157 mAh g^−1^ after 200 cycles at a high current density of 100 mA g^−1^, with a capacity retention rate of 87.73%. It improves the problem of fast capacity decay of bulk phase materials and has a broad prospect for the option of suitable anode materials for sodium-ion battery applications.

## 2. Materials and Methods

### 2.1. Materials

Graphite (2000 mesh) was obtained from Aladdin Industries Ltd.; bacterial cellulose (0.80%) under further purification was purchased from Qihong Technology; analytical grade isopropyl alcohol (IPA) was purchased from Tianli Co., Ltd., Shanghai, China. Sodium was purchased from Shenzhen Youyan Technology Co., Ltd. (Shenzhen, China). Sodium hexafluorophosphate (NaPF_6_) was supplied by Alfa Aesar (purity, ≥99%). Dimethyl carbonate (DMC) and ethylene carbonate (EC) was provided by Sigma-Aldrich. The current collector aluminum foil was supplied by Hefei Kejing Material Technology Co., Ltd., Hefei, China.

### 2.2. Preparation of Bacterial Cellulose/Graphene/Isopropanol Composites

Firstly, 37.5 g (0.8%) of bacterial cellulose, a certain amount of isopropanol solution, and water ($V_{\mathrm{IPA}}$:$V_{H_{2}O}$ = 1:1) were mixed and ultrasonicated in a bath sonicator for 30 min to gain a transparent BC dispersion. An amount of 1.0 g of graphite was added to the above dispersion and then subjected to ultrasonication at 450 W for 4 h using a tip sonicator. In order to prevent the unexfoliated graphite, the suspension was centrifuged (4500 rpm, 45 min), firstly, at 9000 rpm for 25 min to better deposit large flakes and multilayer graphene in the next step. The supernatant was collected and stored for further use. The obtained liquid exfoliated graphene (LEGr) suspension was then freeze-dried to obtain BC-graphene composites, which were named BC/LEGr-I. For comparison, the BC/graphene composites obtained by LPE using water as solvent were prepared and named BC/LEGr. Additionally, the BC/G and BC/Gr negative electrode materials were collected by mixing raw graphite (G) and graphene by Hummers method (Gr) with BC at the same mass ratio as BC/LEGr-I, respectively. The freeze-dried samples were placed in a tube furnace and slowly heated to 1000 °C at a reaction heating rate of 2 °C/min and annealed for 2 h. The carbonized BC/LEGr-I (termed as CBC/LEGr-I) negative electrode composites were obtained. A similar procedure was performed to prepare CBC/LEGr, CBC/LEGr-I, CBC/Gr, and CBC/G, respectively.

### 2.3. Material Characterization

A high-resolution field emission scanning electron microscope (HRFE-SEM, S-4800, Hitachi, Japan) and a transmission electron microscope (TEM, Tecnai G2 F20, FEI, Hillsboro, OR, USA) were used to observe the morphology of the samples. The structure of prepared nanocomposites was characterized by Fourier transform infrared method (FTIR, VECTOR-22, Ettlingen, Germany). The crystal structure of the samples was characterized by X-ray diffraction (XRD, MiniFlex 600, Rigaku, Japan, Cu Kα, 2θ range of 5°–80°) using Cu Kα rays in the 2θ range of 5°–80°. The absorption spectra of the graphene dispersion were obtained with an Ultraviolet-visible Spectrometer (UV-Vis, UV-2600, Shimadzu, Japan). Raman spectroscopy (Raman, THEM DXRxi, Thermo Fisher Scientific, Waltham, MA, USA) was adopted to evaluate the molecule structure, especially defects or disorders induced by the structure of nano carbon materials with a 532 nm laser. Finally, surface morphology of the sample was estimated by atomic force microscopy (AFM, SPI3800N/SPA400, Tokyo, Japan).

### 2.4. Electrochemical Test

The slurry consists of active materials, conductive carbon, and polymer binder N-methyl-pyrrolidone (NMP), which was prepared (mass ratio 8:1:1). It was painted on clean aluminum foil and then thoroughly vacuum-dried to obtain working electrodes with the quantitative load of about ~0.8 mg cm^2^. Electrochemical tests were performed by assembling the coin cell (CR-2032) in an argon-filled glove box. The electrolyte was prepared with a solution of 1 M NaPF_6_ in dimethyl carbonate (DMC) and ethylene carbonate (EC) (1:1, by volume). Charge/discharge tests were conducted by a battery test system (LAND-CT3001A, Wuhan Landian Electronic Co., Ltd., Wuhan, China). in the voltage range of 0.001–2.5 V. Cyclic voltammetry (CV, 0.01–2.5 V at 0.1 mV s^−1^) and electrochemical impedance (EIS, 0.1–100,000 Hz at 5 mV) were performed on an electrochemical workstation (PARSTAT3000A-DX, Ametek, Berwyn, PA, USA). CV was performed in the voltage range of 0.01–2.5 V with a scan rate of 0.1 mV s^−1^. EIS was performed at an amplitude of 5 mV with a frequency range of 0.1–100,000 Hz.

## 3. Results and Discussion

### 3.1. Morphological and Structural Characterization

**Figure 1:**
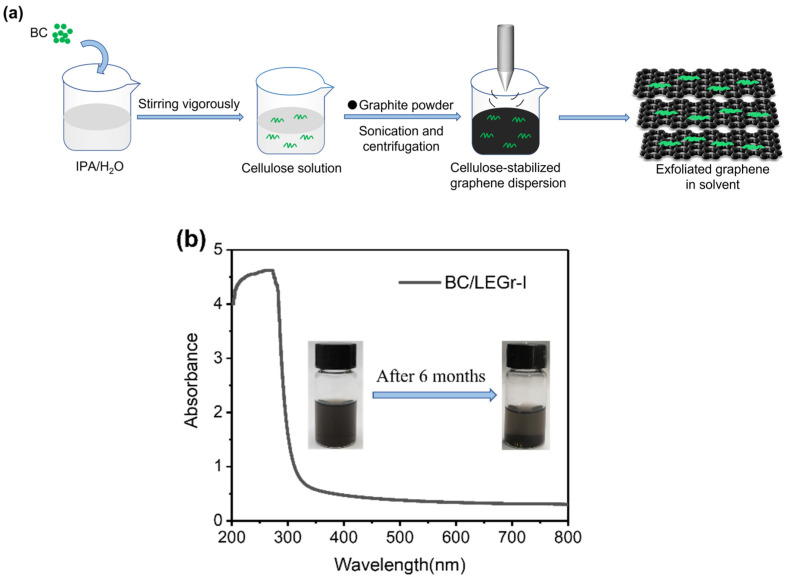
(**a**) Schematical illustration of BC-stabilized few layer graphene dispersions through BC/solvent assisted liquid phase exfoliation (LPE). (**b**) UV-Vis spectra of BC/LEGr-I dispersion and inset of BC/LEGr-I dispersion standing for six months.

The procedure for preparing BC-stabilized few layer graphene dispersions through BC/solvent assisted liquid phase exfoliation (LPE) is represented in Figure 1. The precursor dispersion was obtained by dispersing BC in deionized water and isopropanol mixed solution, and then addition of graphite powder was added. Under vigorous sonication, the formed LEGr can be stabilized by BC through non-covalent intermolecular forces to obtain steady BC/LEGr-I composite dispersion (Figure 1a) [20]. After standing for six months, BC/LEGr-I dispersion remains stable without any significant precipitation. (Figure 1b inset)

The concentration of LEGr nanosheets were estimated by UV-Vis spectra as previously reported [27]. There is an obvious absorption peak at 275 nm, indicating that few-layered graphene can be exfoliated from bulk graphite and stabilized by BC/solvent molecules under ultrasonication [19] (Figure 1b). Meanwhile, the total concentration of BC/Gr composite was determined gravimetrically by weighting the solid from BC/Gr dispersion through vacuum drying at 60 °C for 5 h. Then, the BC/LEGr concentration was calculated as follows:

(1) $$c_{1}=\left(m_{2}-m_{1}\right)⁄V$$

*m*_1_ and *m*_2_ are the mass of the empty vial and vial with dried BC/LEGr, respectively, and *V* is the volume of the BC/LEGr dispersion. We calculated the concentration $c_{2}\;$of the prepared LE graphene based on UV-Vis results using Lambert-Beer law [28]:

(2) $$A=\alpha c_{2}l$$

where *A* represents the absorbance, *α* represents the absorbance coefficient of graphene (mL mg^−1^ m^−1^), and l represents the width of the cuvette (cm). According to previous research [29], the absorbance coefficient *α* of the exfoliated graphene suspension was 2460 mL mg^−1^ m^−1^ at 660 nm. Therefore, the concentration of LEGr was calculated to be 0.1317 mg mL^−1^ with 2.63% yield. Based on the total mass of BC/LEGr-I calculated by Equation (1), the mass ratio of BC/LEGr-I is 4:1.

**Figure 2:**
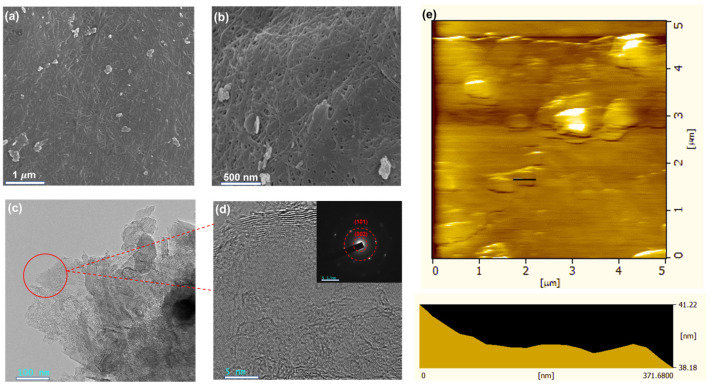
(**a**,**b**) SEM morphology of CBC/LEGr-I; (**c**,**d**) TEM and SAED pattern images of CBC/LEGr-I; (**e**) AFM images and corresponding height profiles of BC/LEGr-I taken along the lines are shown in the insets.

The specific morphology of the LEGr nanosheets was exhibited by SEM, TEM, and AFM in Figure 2. The LEGr nanosheets could be clearly seen after pyrolysis compared to CBC/G and CBC/Gr, and they were embedded in the BC three-dimensional network structures, with the dimension ranging from 250 nm to 500 nm (Figure 2a,b and Figure S1a,b). The retained BC network after pyrolysis can help the diffusion of electrolyte in the material. TEM images confirmed the successful exfoliation and formation of LEGr nanosheets (Figure 2c,d). The strong electron beam can partially penetrate the ultrathin graphene nanosheet layer, and thus a translucent effect can be found in the TEM images at the edge part of the composite. The graphene folds also emerge, which can improve the electrochemical activity and electrochemical properties. Meanwhile, the crystal structure of LEGr nanosheets can be examined by SAED in the inset of Figure 2d. The corresponding electron diffraction pattern of CBC/Gr in Figure S1d shows the typical hexagonal shape of graphene. From the SEM and TEM morphology were observed, in Figure S1a,c, it can be seen and CBC/G presented a totally different structure, which was composed of multiple lamellar layers stacked together (Figure S1a,c). The CBC/LEGr-I displays similar micromorphology with CBC/Gr (Figure S1b), demonstrating that BC-assisted LPE was able to produce graphene with high quality comparable to that of the chemical oxidation-exfoliation method. The AFM image of obtained LEGr also indicates that graphene sheets were successfully exfoliated from the pristine graphite (Figure 2e). The AFM images and the height profile (Figure 2e) show that the thickness of the LEGr nanosheets is about 3.04 nm [30], and the lateral dimension is about 250 nm to 500 nm. Meanwhile, considering the inevitably restacking of the LEGr nanosheets due to evaporation of the solvent during sample preparation, the actual number of layers should be less than the observed value.

**Figure 3:**
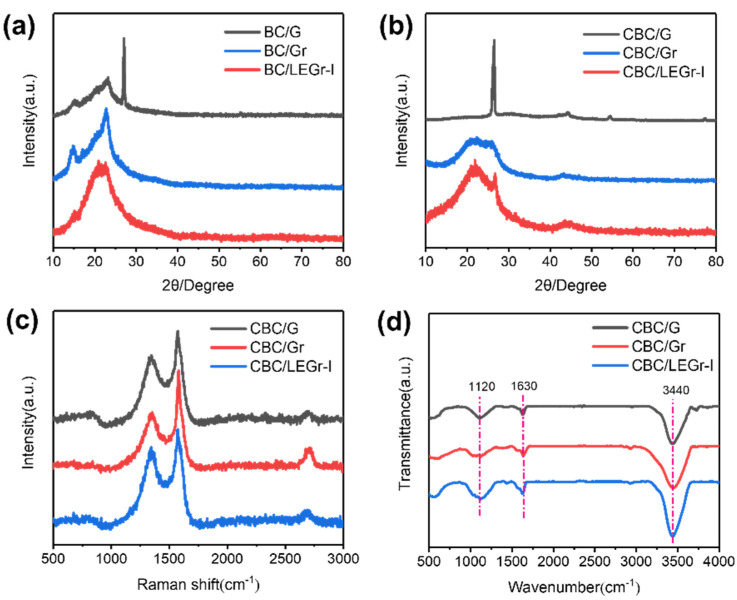
(**a**) XRD patterns of BC/G, BC/Gr, and BC/LEGr-I. (**b**) XRD patterns, (**c**) Raman-spectroscopies and (**d**) FT-IR spectrum of CBC/G, CBC/Gr, and CBC/LEGr-I.

The XRD patterns of BC/G, BC/Gr, and BC/LEGr-I composites are shown in Figure 3a. BC/G showed characteristic peaks at 15°, 23°, and 26°, respectively. The high-intensity peak at 26° is associated with the (002) crystallographic plane of graphite, and the broad diffraction peak at 23° is associated with the (200) crystallographic plane of nanocellulose structure. The (002) peak at 26° in BC/Gr, BC/LEGr-I becomes weaker, or even disappears, because of overlapping with nanocellulose peaks, especially for that in BC/LEGr-I. The changes in peak intensity indicate the decrease in carbon crystallinity due to the formation of few layered graphene sheets after exfoliation [31]. The XRD patterns of the composite materials after carbonization are displayed in Figure 3b. The CBC/Gr and CBC/LEGr-I composites both have carbon characteristic peaks at 23° and 26°, which represented carbonized nanocellulose and graphite, respectively. The broad diffraction peak displayed at 43° is attributed to the (101) planes of graphene structure [32,33]. To point out, the (002) graphite peak of CBC/LEGr-I is still broaden and weak even after thermal anneal, which is due to the smaller size, the decrease in the integrity of the crystal structure, and increase in disorder of the exfoliated graphene sheets. In contrast, the abovementioned peak is sharp and strong in CBC/G, which covered other carbon peaks from CBC.

The Raman spectra of BC/G, BC/Gr, and BC/LEGr-I are shown in Figure S2. The D band at 1350 cm^−1^ demonstrates the structural defects caused by sp^3^ hybridization and oxygen-containing functional groups, while the G peak at 1580 cm^−1^ represents the stretching movement of sp^2^ hybridized carbons [32,34,35]. The two-dimensional peak at 2700 cm^−1^ also corresponds to the disorder of the structure, and it is normally an indicator of the number of graphene layers [36]. The degree of defects in a sample can also be defined by calculating the ratio of the D peak intensity to the G peak intensity, representing an increase in edge structure [37]. The formation of the BC-LEGr complex is confirmed by the characteristic Raman vibrational peaks of BC in addition to the D, G and 2D peaks of graphene as seen in Figure S2. The Raman spectra of the composite after carbonization are displayed in Figure 3c. After carbonization, the D band intensity increases significantly, and the I_D_/I_G_ increases to 0.730, 0.566, and 0.805 for CBC/G, CBC/Gr, and CBC/LEGr-I, respectively. The increased number of defects and edges in the nanosheets, as indicated by higher I_D_/I_G_ ratio, creates additional sodium ion storage sites. The increased disorder structure in CBC/LEGr-I composites revealed by Raman spectra is also consistent with the XRD results, which can be explained by better exfoliation, and suggests better electrochemical performance. Meanwhile, compared with CBC/G, the evolution of two-dimensional bands shape in CBC/LEGr-I indicates that the graphene layers are exfoliated from the bulk graphite, and its position and shape are confirmed as few-layer graphene [36]. The FT-IR spectra of CBC/G, CBC/Gr, and CBC/LEGr-I are demonstrated in Figure 3d. The strong peak at 1630 cm^−1^ is attributed to the presence of sp^2^-conjugated graphene sheets. The absorption peak at 3440 cm^−1^ is accompanied by the vibration of residue -OH containing groups on the pyrolyzed BC backbone. By comparing the IR curves of the three samples, it is shown that the addition of BC does not affect the crystal structure of LEGr, thus demonstrating that bulk graphite is successfully exfoliated into graphene with BC/solvent assistance, as confirmed by AFM previously.

### 3.2. Electrochemical Properties

**Figure 4:**
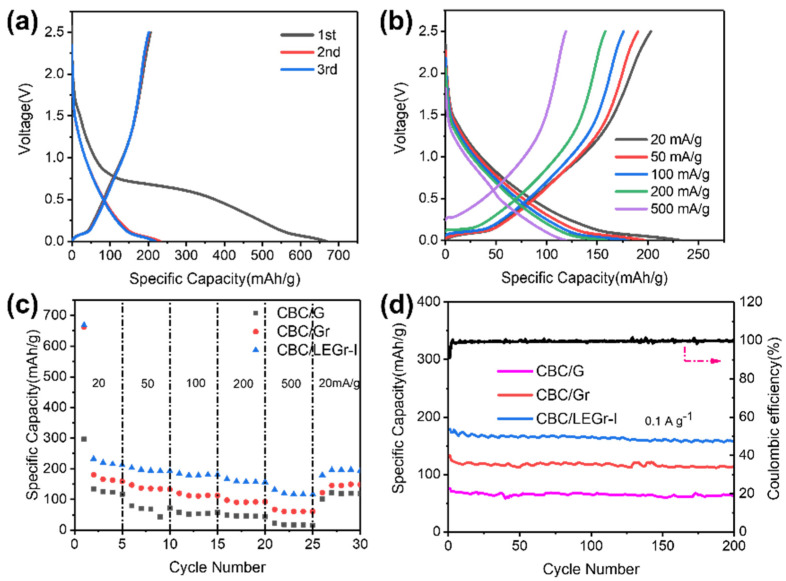
Galvanostatic charge/discharge curves of CBC/LEGr-I (**a**) for first three cycles at 0.02 A g^−1^ (**b**) at different rates. (**c**) Rate capacity at different rates and (**d**) cycling stability at 0.1 A g^−1^ of CBC/G, CBC/Gr, and CBC/LEGr-I.

The electrochemical performance of coin cells assembled with CBC/G, CBC/Gr, and CBC/LEGr-I composite negative electrodes, as well as sodium metal positive electrode using ester-based electrolyte, were investigated. Figure S3a,band Figure 4a present the first three charge/discharge profiles of CBC/G, CBC/Gr, and CBC/LEGr-I at a current density of 20 mA g^−1^, respectively. The curves mainly are comprised of two distinct voltage regions. On the one hand, the slope region above 0.1 V corresponds mainly to the adsorption of Na^+^ on surface active sites. On the other hand, and the plateau area below 0.1 V mainly corresponds to the insertion of Na^+^ between carbon layers or the filling of Na+ into nanomoles [38]. The proportion of the slope area exceeds that of the plateau area for the three electrodes, which account for 64.96%, 62.17%, and 68.58% of the overall reversible discharge capacity correspond to CBC/G, CBC/Gr, and CBC/LEGr-I, respectively. The higher slope capacity in CBC/LEGr-I indicates that more surface-active sodium storage sites were provided by LEGr nanosheet assembly. The initial charge/discharge capacities and ICE are 114/296 mAh g^−1^ (ICE: 38.33%), 158/662 mAh g^−1^ (ICE: 23.78%), and 206/669 mAh g^−1^ (ICE: 30.84%) for CBC/G, CBC/Gr, and CBC/LEGr-I at a current density of 20 mA g^−1^, respectively. CBC/LEGr-I electrode presents much higher reversible capacity compared to CBC/G and improved ICE compared to CBC/Gr. As discussed in Figure 4c, the rate performance of the three composite electrodes were compared at current densities from 20 mA g^−1^ to 500 mA g^−1^. The discharge specific capacities of CBC/LEGr-I were 233, 203, 185, and 167 mAh g^−1^ at current densities of 20, 50, 100, and 200 mA g^−1^, respectively. Moreover, discharge specific capacity of 132 mAh g^−1^ can still be maintained at 500 mA g^−1^. Furthermore, the specific capacity of the CBC/LEGr-I electrode can almost fully recover to 180 mAh g^−1^ when the current density returned to (20 mA g^−1^), indicating its high stability and excellent rate performance at the wide range of current densities. The coulombic efficiency (CE) was 30.84% in the initial cycle, and then it rapidly increased to 99.05% in the subsequent cycles. Due to the formation of SEI, this caused the unsatisfied initial coulombic efficiency (ICE) of the CBC/LEGr-I electrode. The galvanostatic charge/discharge profiles of the CBC/G, CBC/Gr, and CBC/LEGr-I at various rates from are displayed in Figure S3c,d and Figure 4b. Figure S4a exhibits the first three galvanostatic charge/discharge profiles at 20 mA g^−1^ of CBC/LEGr, which were obtained by BC-assisted exfoliation in water. The first discharge and charge capacities are 458 and 116 mAh g^−1^ with a low ICE of (25.44%). The deteriorated performance compared to CBC/LEGr-I revealed that existence of co-solvent was also essential for LPE. As shown in Figure 4d, CBC/LEGr-I has the highest reversible capacity and better cycling stability compared to CBC/G, CBC/Gr, and CBC/LEGr-I, the latter of which has the better cycling stability and highest reversible capacity. The reversible capacity of CBC/LEGr-I reached 157 mAh g^−1^ after 200 cycles, with a capacity retention rate of 87.73%. By comparison, the reversible capacities of CBC/G and CBC/Gr were only 32.4 and 115 mAh g^−1^ under the same conditions, which are due to the higher graphitization degree (G) or lower exfoliation degree (Gr). The stacking of graphene/graphite layer affects the rapid diffusion of sodium ions. In other words, only a small fraction of sodium ions can be stored in the SIBs, most of which form a plating layer on the graphite surface, providing fewer adsorption and desorption sites [39]. Owing to better graphene quality by BC/co-solvent exfoliation, CBC/LEGr-I with abundance of defects and Na^+^ storage active sites presented the highest reversible capacity.

**Figure 5:**
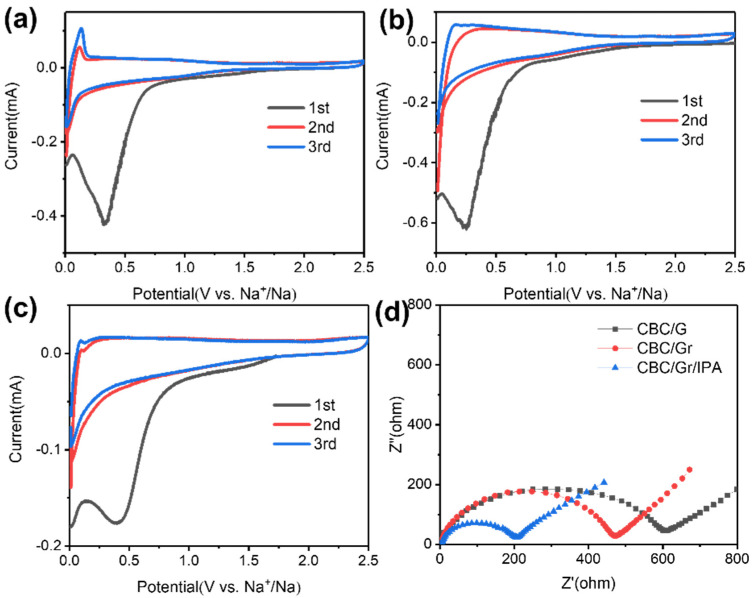
(**a**–**c**) CV curves of 1st, 2nd, and 3rd cycles at 0.1 mV s^−1^ and (**d**) Nyquist plots of CBC/G, CBC/Gr, and CBC/LEGr-I.

Figure 5a–c exhibits the representative first three cyclic voltammogram (CV) curves of the CBC/G, CBC/Gr, and CBC/LEGr-I at scan rate of 0.1 mV s^−1^ between a range of 0.001–2.5 V. The reduction peak at 0.33 V is due to irreversible reaction of the SEI film formation on the electrode surface in the initial cycle [40,41]. The remarkable reduction peak at 0.001–0.16 V can be assigned to sodium ions adsorption and intercalation/nanopore filling [42]. The oxidation peak located at 0.16 V reveals the desorption of sodium ions, which almost overlaps in the second and third cycles. A long slope is observed between 1.6 and 0.01 V at the cathode and a broad anodic peak at 0.1 V at the anode, indicating a good stability and reversibility of the sodium ion during de-intercalation/desorption. To further reveal the improvement of sodium storage property, we performed electrochemical impedance spectroscopy (EIS) tests on all samples. The depressed semicircle section and straight line of the Nyquist plots for all composite electrodes correspond to the high frequency/medium frequency region and the low frequency region, respectively (Figure 5d). The high-frequency semicircle corresponds to the charge-transfer resistance (Rct) and the SEI, in contrast to the slope in the low-frequency region, which indicates the diffusion resistance to ion transfer, that is, the Warburg impedance of Na^+^ diffusion in hard carbon. It is clear that CBC/LEGr-I has the smallest semicircle diameter, i.e., the smallest resistance. This indicates that the BC/solvent-assisted, liquid-phase exfoliated graphene composites are better able to facilitate sodium ion and electron transfer and improve the conductivity with the best sodium storage performance.

## 4. Conclusions

In summary, we demonstrated an effective and simple strategy to achieve green and efficient preparation of graphene (LEGr) from raw graphite (G) via bacterial cellulose (BC)/solvent-assisted direct liquid-phase exfoliation. Then, CBC/LEGr-I composites were obtained after carbonization and showed superior electrochemical properties than CBC/G and CBC/Gr (graphene via Hummers method). As a green dispersant/stabilizer, the BC helps to exfoliate raw graphite along with solvent, so as to produce few layered graphene comparable to that of Hummers method. The addition of BC not only prevented the re-stacking of the exfoliated partial graphite flake layers, but also acted as a precursor for carbon nanofiber to further enhance the performance. The CBC/LEGr-I has a good cycle life, with a specific capacity of 157 mAh g^−1^ and a capacity retention rate of 87.73% after 200 cycles, which outperformed the CBC composite electrode with graphene from chemical oxidative-exfoliation. Such an improved LPE method can be used to effectively produce graphene-based carbon composite materials for SIB.

## Data Availability

Not applicable.

## Supplementary Materials

The following supporting information can be downloaded at: https://www.mdpi.com/article/10.3390/polym15010203/s1, Figure S1: Characterization; Figure S2: Characterization; Figure S3: Electrochemical Measurement; Figure S4: Electrochemical Measurement.
