# Supplementary material for: Graphene Composite via Bacterial Cellulose Assisted Liquid Phase Exfoliation for Sodium-Ion Batteries
Source: Polymers (Basel). 2022 Dec 31;15(1):203. doi: 10.3390/polym15010203 (PMC9824451; doi:10.3390/polym15010203)
Supplement: Supplementary file 1 [file polymers-15-00203-s001.zip › polymers-2100375-supplementary.pdf]

Graphene composite via bacterial cellulose assisted liquid phase exfoliation for sodium-ion batteries Supplementary Information

Characterization

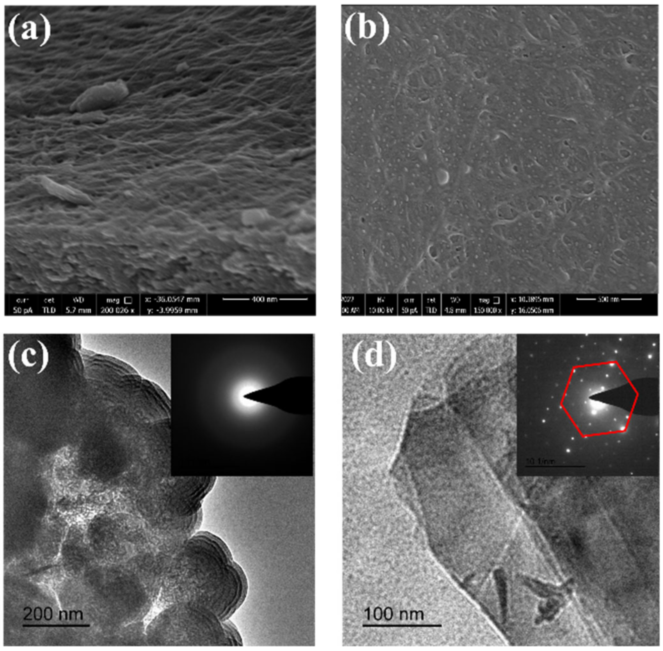

Figure S1. SEM images of (a) CBC/G and (b) CBC/Gr; TEM and SEAD images of (c) CBC/G and (d) CBC/Gr (region displaying the hexagonal lattice and corresponding SAED pattern).

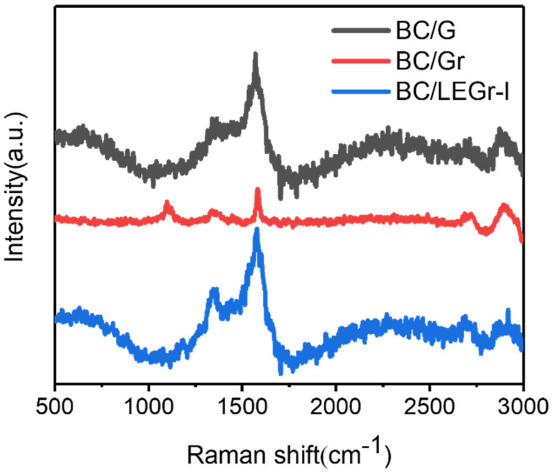

Figure S2. Raman-spectroscopies of BC/G, BC/Gr and BC/LEGr-I.

Electrochemical Measurements

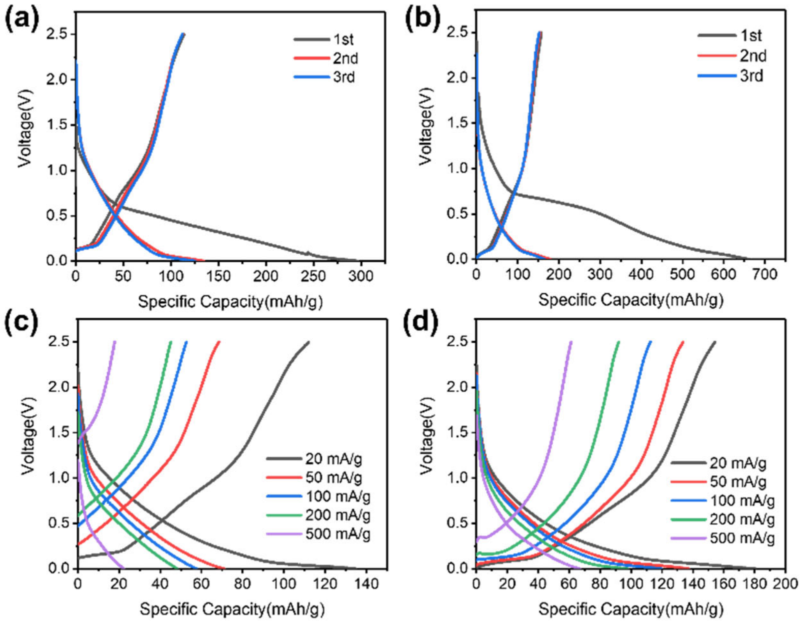

Figure S3. Galvanostatic charge/discharge curves for first three cycles at 0.02 A g-1 of (a)CBC/G and (b)CBC/Gr. Galvanostatic charge/discharge curves at different rates of (c) CBC/G and (d)CBC/Gr.

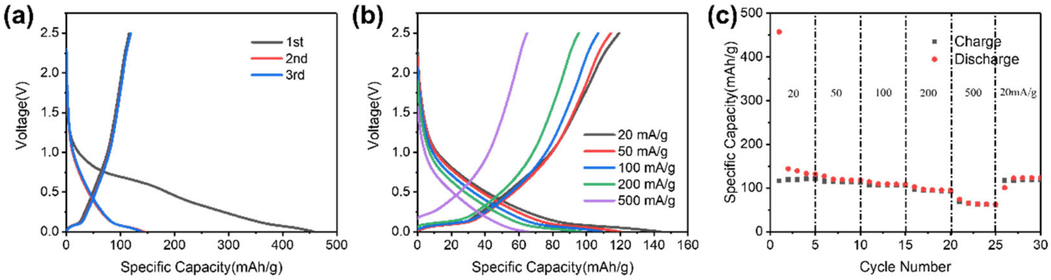

Figure S4. Galvanostatic charge/discharge curves of CBC/LEGr (a) for first three cycles at 0.02 A g-1 (b) at different rates and (c) rate capacity at different rates.
